# Fear of prognosis? How anxiety, coping, and expected burden impact the decision to have cytogenetic assessment in uveal melanoma patients

**DOI:** 10.1007/s00520-022-07006-5

**Published:** 2022-03-31

**Authors:** Johannes Gollrad, Nevenka Korpusik, Christopher Rabsahl, Dirk Boehmer, Angela Besserer, Ulrike Grittner, Alexander Boeker, Ulrich Keilholz, Antonia Joussen, Volker Budach, Ute Goerling

**Affiliations:** 1 Department of Radiation Oncology, Charité – Universitätsmedizin Berlin, Berlin, Germany; 2 Institute of Biometry and Clinical Epidemiology & Berlin Institute of Health at Charité, Charité – Universitätsmedizin Berlin, Berlin, Germany; 3 Department of Ophthalmology, Charité – Universitätsmedizin Berlin, Berlin, Germany; 4 Charité Comprehensive Cancer Center, CCCC, Charité – Universitätsmedizin Berlin, Berlin, Germany; 5 Department of Psycho-Oncology, CCCC, Charité – Universitätsmedizin Berlin, Berlin, Germany

**Keywords:** Cytogenetic testing, Uveal melanoma, Anxiety, Cancer prognosis, Proton therapy

## Abstract

**Background:**

Cytogenetic testing (CGT) in uveal melanoma patients reveals prognostic information about the individual risk of developing distant metastasis with dismal prognosis. There is currently no medical intervention strategy with proven effect on the prognosis, rendering the result of the cytogenetic testing purely informative. We explored patients’ socio-demographic backgrounds, psychological preconditions, coping strategies, external influences, and concerns about “knowing their fate” to study their possible interactions with decision-making for CGT.

**Methods:**

Uveal melanoma patients were asked to complete questionnaires on their interest in undergoing CGT for prognostication and the factors influencing their decision. Data were collected on socio-demographics, baseline anxiety (GAD-7), depression (PHQ-9), coping strategies (Brief COPE), and assumed future concerns regarding the CGT result. Data were analyzed by using multiple ordinal logistic regression and exploring estimated marginal effects.

**Results:**

Questionnaires were returned by 121 of 131 (92.4%) patients. Fifty-two patients (43%) had no interest in CGT, 34 (28.1%) were undecided, and 35 (28.9%) were interested. We observed no significant differences regarding age, sex, partnership, education, occupation, baseline anxiety, or depression. Decision-making favoring CGT was influenced by the treating physicians, internet resources, and level of baseline anxiety. Patients were likely to reject CGT when they worried that “knowing the result will have an unintended influence” on their life.

**Conclusion:**

Decision-making about CGT for prognostication in uveal melanoma is burdensome to many patients and in general not guided by medical advice regarding further treatment and screening procedures. The psychological impact of the decision is therefore unique and requires careful support by psycho-oncologists considering the patient’s fears and expectations.

## Introduction

Uveal melanoma is a rare disease with an incidence of up to 8.6 per 1 million population in Europe [1]. Although eye-preserving local treatment of the primary tumor—for example, plaque or proton beam therapy—has been shown to be highly effective, the disease has a dismal prognosis when distant metastases occur [2, 3]. The results of cytogenetic testing (CGT) of tumor material at the time of initial diagnosis are a major determinant in assessing the probability of future distant metastasis [4]. In particular, the presence of monosomy 3 in enucleated uveal melanoma patients has been associated with a more than fivefold increase in risk for developing metastasis and subsequent death compared with disomy 3 after a median follow-up time of 5.2 years (overall survival 13.2% vs 75.6%)[5].

However, CGT in uveal melanoma differs substantially in several aspects from most other tests established in the context of cancer. While, for example, BRCA testing of breast cancer patients affects the further therapeutic strategy or screening procedures for patients as well as family members, genetic testing of uveal melanoma patients has no consequences regarding further therapy or follow-up [6, 7]. In addition, CGT requires a specific tumor biopsy, which is not standard in uveal melanoma where histological confirmation is not essential for diagnosis. This means that CGT in uveal melanoma provides purely prognostic information to the patient and potentially useful scientific information to cancer research.

Knowing their fate could have significant implications for patients in terms of their psychological state and future life planning [8–10]. Some authors state that this information could be burdensome to patients and may be reflected in higher scores for depression, higher distress, and lower quality of life in patients with monosomy 3, as reported by Hope-Stones et al. and Reimer et al. [9, 11, 12]. Other studies found that test results have only a minor impact on anxiety and depression, and largely show mental QOL comparable to that of an age-matched healthy norm population [8, 13]. Lieb et al. prospectively investigated 63 patients opting for CGT and showed that the perceived risk of developing metastasis increased in patients after receiving a poor prognosis and decreased in patients with good prognosis [10]. However, the same study observed that anxiety, depression, general distress, and fear of progression declined equally in all patients after primary treatment, regardless of their prognosis or their decision about undergoing CGT. Interestingly, a qualitative study by Cook and colleagues, who thoroughly interviewed 22 patients before and after CGT, revealed that the CGT result was experienced in a somehow contradictory manner: patients with good prognosis did not find the reassurance they expected and patients with poor prognosis turned the significance of this “knowledge” into a suspected but unproven health benefit [9].

To date, little is known about patients’ motivation for testing and how patients reflect on their decision. Arguments advocating for CGT in uveal melanoma include a more pronounced sense of control, autonomy, hopefulness, and better life planning [9, 10, 13, 14]. However, Deber and colleagues showed that most patients prefer a more passive role and show little strive for autonomy in the decision-making process [15]. Cook et al. reported that patients’ decisions seemed mostly not self-determined but strongly influenced by their relationship with a trusted, caring medical practitioner [9]. Moreover, patients in that study expected that a poor prognosis would influence their life planning.

Patients’ interest in receiving prognostic information could depend on many factors. Previous research has shown that general distress, degree of social support, and perceived risk of developing metastasis may each have an impact [10]. In addition, the primary treatment method seems to play a role in decision-making. For example, Lieb et al. showed that patients undergoing enucleation were more likely to be interested in genetic analysis, compared with patients receiving plaque therapy [10]. In this study, only 6 patients received proton therapy. In general, previous investigations into this issue have included no or only a few patients undergoing proton therapy for uveal melanoma.

While previous psycho-oncological literature primarily focused on the impact of the testing result on the patient’s psychological well-being or decision regret over time, to date, we know little about the determinants affecting the decision to test [8–10, 12, 13]. The present study was designed to prospectively address the process of decision-making itself, irrespective of the testing result. We focused on investigating the underlying extrinsic and intrinsic preconditions, guiding the patient’s decision on CGT. To this end, we explored the impacts of socio-demographic background, anxiety, depression, and coping strategies on the decision-making process for CGT in uveal melanoma patients treated with proton therapy. In addition, we aimed to gain a better understanding of the patients’ motivations behind their decision by addressing the patients’ individual concerns regarding “knowing the prognosis” and its assumed impact on future life and planning.

## Methods

Between May 2019 and January 2020, 183 patients diagnosed with non-metastatic uveal melanoma and in preparation for primary proton treatment were screened for participation in our prospective quality-of-life program. The study was conducted according to the Declaration of Helsinki and approved by the local ethics committee.

### Inclusion and exclusion criteria

Patients were considered eligible if they met the following inclusion criteria: primary diagnosis of non-metastasized uveal melanoma, no previous tumor-directed treatment and informed consent obtained before radiotherapy. Exclusion criteria were known metastatic disease of any tumor or insufficient German language skills.

### Procedure

After giving informed consent, all participants were provided with standardized written information about optional CGT. In this information sheet, we emphasized that CGT aims to provide more precise prognostic information about the patient’s individual risk of developing metastasis with subsequently fatal outcome, irrespective of successful primary treatment. Patients were informed that the CGT result, according to actual guidelines and best clinical practice, would not change their medical treatment nor improve their outcome. All questionnaires were completed and returned within 5 to 12 days prior to the start of proton therapy. Importantly, the expressed interest in CGT was completely independent of any patient’s decision regarding an actual and imminent intervention associated with CGT.

### Questionnaires

Patients completed questionnaires on socio-demographics, interest in CGT (Likert scale, seven grades), and expected future life changes in case of “knowing” the CGT result (Likert scale, Table 3). Patients were asked how strongly their decision was influenced by treating physicians, family members, friends, internet resources, and their financial situation. In addition, we asked patients to rate on a scale of 0 (not informed) to 10 (very well-informed) how well-informed they felt about the disease and therapy. Furthermore, we collected data on the psychological conditions of our patients, using validated questionnaires for generalized anxiety disorder (GAD-7) [16], depression (PHQ-9) [17], and coping strategies (Brief COPE) [18].

### Rationale for variable selection

The self-reported influence of professionals, relatives, and friends on CGT decision-making was assessed in order to explore the level of autonomy of the patient’s decision, as questioned by several authors, previously [9, 14, 15]. Anxiety and depression are commonly described issues during diagnosis and follow-up of uveal melanoma patients [10, 13, 19–22]. As both conditions have been frequently reported to peak at the time of diagnosis and slowly diminish over time, anxiety and depression were assumed to influence CGT decision and therefore analyzed in this study. Although decision-making on CGT has been repeatedly described difficult and burdensome for many patients, to our knowledge, coping strategies have not been previously addressed in the context of CGT. By including the Brief COPE questionnaire into our analyses, we expected a better understanding of the potential impact of behavioral factors on decision-making.

### Statistics

Statistical evaluation was performed using R (version 4.0.5) with the packages *MASS* and *sjPlot* [23–25]. All patients were grouped by “interested in” (1–2), “undecided” (3–5), or “not interested” (6–7) in CGT, according to Likert scale–based scoring. Differences between subgroups were analyzed using Kruskal–Wallis and Mann–Whitney *U* tests. The correlation analyses were performed using Spearman’s rank correlation coefficient. Ordinal logistic regression modeling was used to analyze characteristics associated with decision-making regarding CGT. Interactions between selected characteristics were also studied. Estimated marginal effects and 95% confidence intervals (CIs) are reported. Additionally, we calculated the standardized mean difference (*smd*) or average *smd* for quantifying group differences. The *smd* is a standardized effects size that is independent of the sample size. It is Cohen’s *d* in the case of comparing two groups in a continuous measure. We used the calculation of the *smd* as implemented in the R package tableone with extensions of the *smd* for nominal data[26].

## Results

A total of 154 patients could be contacted and screened for participation in the study. Of these patients, 10 (6.5%) were excluded for insufficient German-language skills, and 7 (4.5%) for tumor recurrence, while 6 (3.9%) patients withdrew their consent for participation. Finally, 131 patients participated in the study, of whom 121 (92.4%) answered the question about interest in cytogenetic testing of the tumor. The mean age was 59 years (range = 20–84 years, *SD* = 14, 61 of 121 [50.4%] were male). Fifty-two patients (43%) reported having no interest in cytogenetic testing. A smaller number of patients were interested (*n* = 35, 29%) or undecided (*n* = 34, 28%). The socio-demographics and tumor characteristics of the patients are shown in Table 1.

**Table 1:** Sociodemographic data and tumor characteristics for all patients and subgroups with different interests in genetic testing. Interest scale score 1–2 = interested in genetic testing; score 3–5 = undecided; score 6–7 = not interested; shown are mean values with standard deviation (*SD*) for age and tumor characteristics. *P P* value for testing differences between groups using the Kruskal–Wallis test or Chi-squared test (marked with*); *Average smd* average standardized mean difference for quantifying group differences; *AJCC* American Joint Cancer Committee 8^th^ ed

Tumor characteristics and socio-demographics	Interest in genetic testing									
	All patients		Interested		Undecided		Not interested		*P*	Average *smd*
Patients (*n*)	121		35	*29%*	34	*28%*	52	*43%*		
Male	61		19	*31%*	15	*25%*	27	*44%*	0.672	0.14
Female	60		16	*27%*	19	*32%*	25	*42%*		
Mean age (years), *SD*	58.8	13.7	58.3	11.6	57.2	15.4	60.2	13.8	0.593	0.15
AJCC tumor stages										
T1a/c	43		11	*26%*	6	*14%*	26	*60%*	0.219*	0.61
T2a/d	42		13	*31%*	15	*36%*	14	*33%*		
T3a/b	29		9	*31%*	10	*34%*	10	*35%*		
T4a/b	7		2	*29%*	3	*43%*	2	*29%*		
Tumor characteristics										
Tumor prominence (mm)	4.6	*3.1*	4.7	*2.9*	5.5	*3.6*	4.0	*2.8*	0.076	0.33
Tumor base diameter (mm)	15.0	*4.0*	15.6	*4.0*	15.7	*3.5*	14.0	*4.1*	0.067	0.30
Tumor volume (mm^3^)	486	*454*	516	*403*	616	*534*	380	*411*	0.055	0.35
Partnership and household										
Living alone	21		3	*14%*	6	*29%*	12	57%	0.707*	0.49
Living with partner	84		25	*30%*	24	*29%*	35	42%		
Living with partner and kids	12		5	*42%*	3	*25%*	4	33%		
Other*	4		2	*50%*	1	*25%*	1	25%		
Having own kids										
Kids	90		29	*32%*	20	*22%*	41	*46%*	0.047	0.36
No kids	31		6	*19%*	14	*45%*	11	*35%*		
Highest education										
Sec. school (9–10 years)	23		5	*22%*	6	*26%*	12	*52%*	0.614*	0.28
High school/working dipl	63		18	*29%*	16	*25%*	29	*29%*		
University diploma	34		12	*35%*	12	*35%*	10	*35%*		
Occupation										
Employed	66		23	*35%*	19	*29%*	24	*36%*	0.118*	0.64
Unemployed	5		1	*10%*	0	*0%*	4	*80%*		
Retired	45		10	*22%*	12	*27%*	23	*51%*		
Other	5		1	*20%*	3	*60%*	1	*20%*		

### Socio-demographics and tumor characteristics

No significant association between gender, age, partnership, education, or occupation to the probability of interest in genetic testing was observed. Patients with children (*n* = 90) were more likely to have no interest in CGT (*n* = 41, 46%) than those without children (*n* = 11, 35%), while patients with no children (*n* = 31) were more likely to be undecided on CGT (*n* = 14, 45%) compared with patients with children (*n* = 20, 22%; average *smd* = 0.36, *P* = 0.047). Furthermore, patients who were not interested in genetic testing had a smaller mean tumor volume (380 mm^3^, *SD* = 411 mm^3^) compared with patients who were interested (516 mm^3^, *SD* = 403 mm^3^) or undecided (616 mm^3^, *SD* = 534 mm^3^; average *smd* = 0.35, *P* = 0.055).

### Level of feeling informed about the disease and therapeutic options

Patients reported feeling well-informed about their disease and associated therapeutic options (7.2, *SD* = 2.0, scale 0–10, Table 2). The level of feeling informed was not substantially associated with the interest in genetic testing (average *smd* = 0.13).

**Table 2:** Shown are mean values with standard deviation (*SD*) for all patients and subgroups with different interests in genetic testing. Interest scale score 1–2 = interested in genetic testing; score 3–5 = undecided; score 6–7 = not interested. *P P* value for testing differences between groups using the Kruskal–Wallis test; *Average smd* average standardized mean difference for quantifying group differences

	Interest in genetic testing										
	All patients		Interested		Undecided		Not interested		*P*	Average *smd*	
Patients (*n*)	121		35	29%	34	28%	52	43%			
	*Mean*	*SD*	*Mean*	*SD*	*Mean*	*SD*	*Mean*	*SD*			
GAD-7 sum score	5.9	4.3	6.7	5.3	6.5	4.0	5.0	3.7	0.149		0.26
PHQ9 sum score	4.6	4.2	5.2	5.3	4.9	3.7	3.9	3.6	0.671		0.21
Brief COPE (scale 1–4 with 1 = not at all and 4 = strong)											
Self-distraction	2.5	*0.9*	2.4	*0.9*	2.5	*0.8*	2.6	*0.9*	0.653		0.13
Active coping	2.3	*0.8*	2.3	*0.8*	2.3	*0.8*	2.2	*0.8*	0.874		0.08
Denial	1.7	*0.7*	1.7	*0.8*	1.9	*0.8*	1.6	*0.6*	0.197		0.25
Substance use	1.1	*0.3*	1.1	*0.3*	1.2	*0.5*	1.0	*0.2*	0.054		0.33
Emotional support	3.3	*0.8*	3.3	*0.8*	3.3	*0.8*	3.4	*0.8*	0.868		0.07
Instrumental support	2.2	*0.7*	2.1	*0.8*	2.2	*0.8*	2.3	*0.9*	0.441		0.19
Behavioral disengagement	1.5	*0.6*	1.4	*0.5*	1.4	*0.5*	1.6	*0.7*	0.154		0.24
Venting	1.9	*0.7*	2.0	*0.2*	1.9	*0.6*	2.0	*0.7*	0.868		0.08
Positive reframing	2.4	*0.8*	2.4	*0.9*	2.3	*0.7*	2.4	*0.9*	0.710		0.13
Planning	2.3	*0.9*	2.4	*0.9*	2.4	*0.8*	2.2	*0.9*	0.285		0.22
Humor	1.9	*0.3*	2.0	*0.9*	1.7	*0.6*	1.9	*0.9*	0.393		0.23
Acceptance	2.8	*0.9*	2.7	*1.0*	2.6	*0.8*	3.0	*0.8*	0.036		0.38
Religion	1.8	*0.9*	2.0	*1.0*	1.7	*0.8*	1.7	*1.0*	0.176		0.28
Self-blame	1.3	*0.6*	1.5	*0.8*	1.3	*0.5*	1.2	*0.4*	0.065		0.35
Level of feeling informed about disease (scale 0–10 with 0 = not at all informed and 10 = very good informed)											
	7.2	2.0	7.4	2.2	7.0	1.5	7.1	2.0	0.286		0.13

### Anxiety, depression, and coping

The analysis of our data regarding anxiety and depression showed overall low expression for the total sample (mean score 5.9, *SD* = 4.3; and mean score 4.6, *SD* = 4.2; respectively; Table 2). Levels of anxiety and depression were not substantially related to the interest in genetic testing (average *smd* = 0.26 and 0.21, respectively). Regarding coping strategies, we again observed no substantial differences between the subgroups (average *smd* between 0.07 for “emotional support” and 0.38 for “acceptance”).

### Who or what is influencing the patient’s decision on CGT?

**Fig. 1:**
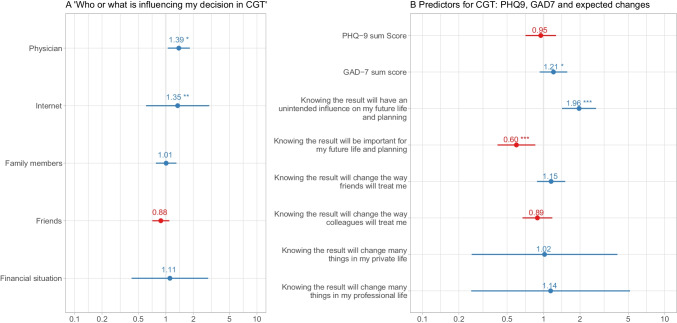
**A** Who or what is influencing my decision on cytogenetic testing? Characteristics associated with interest in CGT in multiple ordinal logistic regression (*n* = 102). *P = 0.009; **P = 0.040. Odds ratios and 95% CIs. **B** Self-reported expected influence of CGT result on future social relations, life planning, level of anxiety (GAD-7), and level of depression (PHQ-9) as characteristics associated with interest in CGT in multiple ordinal logistic regression. (*n* = 105). *P = 0.014; ***P < 0.001. Odds ratios and 95% CIs

When patients were asked who or what may have influenced their decision to undergo genetic testing or not, it was found that patients who were not interested reported being less influenced by family members, friends, medical staff, and online resources, compared with patients who were undecided or favored cytogenetic testing (range = *P* < 0.001 to *P* = 0.0024, average *smd* range = 0.43–0.58). Multiple ordinal logistic regression analyses revealed the influence of physicians (OR 1.39, 95% CI 1.08–1.81, *P* = 0.009) and the internet (OR 1.35, 95% CI 1.10–1.68, *P* = 0.004) as important factors associated with interest in CGT (Fig. 1A).

### Patients’ concerns and expected life changes associated with “knowing” the CGT result

When asked how important the result of genetic testing was for their future life and planning, those patients who were not interested (*M* = 3.2, *SD* = 1.9) reported lower importance than patients who were interested (*M* = 5.1, *SD* = 1.7) or undecided (*M* = 4.8, *SD* = 1.6; *P* < 0.001, average *smd* = 0.72; Table 3). In contrast, when patients were asked whether the result of genetic testing would have an “unintended influence” on their future life, the interested patients assumed that there would be a lesser influence (mean score 3.2, *SD* = 1.9) than that assumed by undecided or uninterested patients (mean score 4.1, *SD* = 2.0 and mean score 4.4, *SD* = 2.2, respectively; *P* = 0.028; average *smd* = 0.40). Major future changes in their private life due to knowing the testing result were expected more frequently by patients interested in genetic testing (M = 3.7; *SD* = 1.9) and undecided patients (M = 4.1; *SD* = 1.9) compared with patients not interested in genetic testing (M = 2.7; *SD* = 1.9; *P* = 0.003, average *smd* = 0.49). In contrast, all patients reported a minor impact of the CGT result in their professional field and regarding their friendships, with no substantial differences between subgroups. Multiple ordinal logistic regression revealed baseline anxiety level (OR 1.21; CI 1.04–1.42; *P* = 0.014), assumed “unintended changes” in the future (OR 0.60; CI 0.45–0.76; *P* < 0.001), and “importance” of the CGT result in future life (OR 1.96; CI 1.49–2.66; *P* < 0.001) as relevant characteristics associated with CGT decision-making (Fig. 1B).

**Table 3:** Shown are mean values with standard deviation (*SD*) for all patients and subgroups with different interest in genetic testing. Interest scale score 1–2 = interested in genetic testing; score 3–5 = undecided; score 6–7 = not interested. *P* level of significance for comparisons between independent subgroups using the Kruskal–Wallis test; *Average smd* average standardized mean difference for quantifying group differences

	Interest in genetic testing											
	All patients			Interested		Undecided		Not interested		P	Average *smd*	
Patients (*n*)	121			35	29%	34	28%	52	43%			
What is influencing my decision for genetic testing (scale 1–7 with 1 = not at all and 7 = strong)												
Family members	2.4		2.1	2.9	2.5	2.8	2.1	1.6	1.5	0.008		0.44
Friends	1.5		1.1	1.8	1.4	1.7	1.4	1.1	0.5	0.002		**0.45**
Medical doctors	2.5		2.0	3.4	2.5	2.8	1.8	1.7	1.5	0.001		0.58
Information resources/Internet	2.4		2.0	3.2	2.2	2.6	1.9	1.6	1.8	0.001		0.52
Financial worries	1.8		1.5	1.9	1.8	2.3	1.8	1.3	1.0	0.012		0.43
Knowing the results of genetic testing will change the way … (scale 1–7 with 1 = not at all and 7 = strong)												
Friends will treat me	2.3		1.8	2.2	1.7	2.7	1.9	2.1	1.7	0.143		0.22
Colleagues will treat me	2.4		1.9	2.3	1.6	2.5	2.1	2.4	1.9	0.974		0.09
Knowing the result of genetic testing I will change many things…. (scale 1–7 with 1 = not at all and 7 = strong)												
In my private life	3.4	2.0		3.7	1.9	4.1	1.9	2.7	1.9	0.003		0.49
In my professional life	2.6	2.0		2.9	1.8	3.0	2.3	2.1	1.8	0.061		0.31
How important is the result of genetic testing for** ….** (scale 1–7 with 1 = not at all and 7 = strong)												
My future life and planning	4.2		2.0	5.1	1.7	4.8	1.6	3.2	1.9	< 0.001		0.72
The result of genetic testing will have an unintended influence** ….** (scale 1–7 with 1 = not at all and 7 = strong)												
On planning my life	4.0		2.1	3.2	1.9	4.1	2.0	4.4	2.2	0.028		0.40

### Correlations between expected life changes and GAD-7 and PHQ-9 sum scores

Further exploratory analyses revealed weak positive correlations between the assumed “unintended influence” of cytogenetic testing on future life and higher baseline anxiety as well as depression (*ρ* = 0.19 and *ρ* = 0.33; Table 4). In contrast, the questionnaire results on “how important” cytogenetic testing would be “for future life and planning” were not associated with anxiety or depression (all *ρ* < 0.03). We also found no direct correlation of anxiety or depression with expected changes in “my private life” or “my professional life” (all *ρ* < 0.11). However, worries about being treated differently by friends and colleagues in case they knew the prognosis were weakly positively related to anxiety and depression (all *ρ* > 0.20).

**Table 4:** Correlations of mean GAD-7 and PHQ9 scores with mean values of Likert scale–based questionnaire results on expected changes in the patient’s future life due to the CGT result. Coefficients and corresponding *P* values were calculated using the Spearman method

Correlations of expected changes on future life with	PHQ-9	GAD-7
I fear knowing the results of my genetic testing will change the way…		
…friends will treat me	0.231 (*P* = 0.013)	0.219 (*P* = 0.018)
…colleagues will treat me	0.285 (*P* = 0.003)	0.208 (*P* = 0.030)
Knowing the result of genetic testing I will change many things…		
…in my private life	0.097 (*P* = 0.302)	0.105 (*P* = 0.263)
…in my professional life	0.107 (*P* = 0.259)	0.029 (*P* = 0.758)
How important is the result of genetic testing for…		
…my future life and planning	0.011 (*P* = 0.904)	0.023 (*P* = 0.803)
The result of genetic testing will have an unintended influence…		
…on planning my life	0.190 (*P* = 0.042)	0.331 (*P* < 0.001)

### Interactions between GAD-7 and life changes expected from knowing the CGT result

**Fig. 2:**
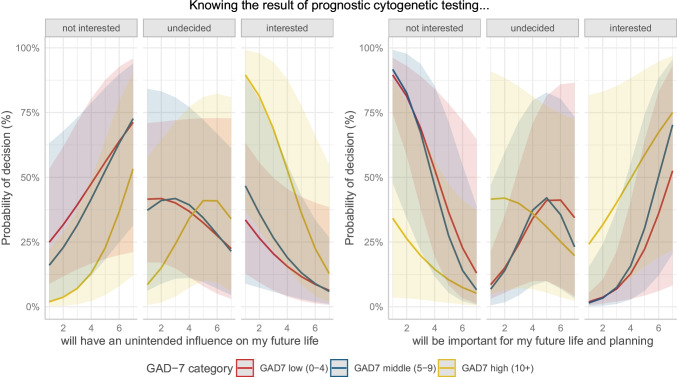
Estimated marginal effects for probability of interest in CGT (not interested, undecided, interested) depending on self-reported assumed influence of the CGT result on future life and planning, grouped by levels of anxiety (GAD-7)

As GAD-7 was found to be associated with CGT decision-making, we further analyzed our data for possible interactions between GAD-7 and life changes expected from knowing the CGT result. Estimated marginal effects analyses identified patients with high baseline anxiety levels as especially vulnerable to fearful expectations regarding their future life and planning. Patients confirming that the CGT result would be important in their future life and planning were the most likely to opt for CGT, with the highest probability in those with high anxiety levels (Fig. 2B). However, the difference in interest in CGT between patients with different anxiety levels was more pronounced in patients reporting less importance of the result in their future life. In addition, patients with high baseline anxiety levels, especially, were more likely to be interested in CGT when they did not expect the result to have an unintended influence on their future life (Fig. 2A). The difference in CGT interest between patients at different anxiety levels was more pronounced in patients who did not fear that the result might have an unintended influence on their future life.

### Correlations to age and sex

Age was seen to be correlated to worries about how friends (*ρ* = –0.33) and colleagues (*ρ* = –0.44) who know the CGT result would treat the patient (*P* < 0.001). In addition, younger patient age was related to the expectation that the testing results “will change many things in my professional life” (*ρ* = –0.45, *P* < 0.001). Older patients saw the genetic testing as less important in “future life and planning” (*ρ* = –0.20, *P* = 0.029). Sex was not substantially associated with any questionnaire results on genetic testing.

## Discussion

In our study, 26.7% of the patients (*n* = 35) were interested in prognostic CGT, which is in line with findings from Lieb et al. (36%) and Beran et al. (38.4%) in patients who finally decided to receive prognostic information[8, 10]. One study observed that 97% of patients opted for CGT [14]. However, those patients based their decision on their expectation of having greater control and better survival due to shortened screening intervals, possibly misinterpreting the given medical information.

In contrast to previous studies, we assessed the interest in CGT during the decision-making process and before definitive local treatment. In addition, by using a Likert scale–based questionnaire, we were able to identify a large group of patients (26%) who were still undecided on this issue. This may underline the complexity of decision-making and the associated insecurities experienced by the patients.

### Who or what is influencing the decision?

We found that our patients favoring CGT were influenced in their decision by the treating physicians and information from internet resources rather than by close relatives or friends. Our results are in line with previous findings that a caring relationship with the treating physician is important when it comes to the decision for CGT [9]. However, Cook et al. revealed that patients described their decision as “fulfilling obligations” to the hospital, other patients, and family members. In contrast, our data underline the extensive need for additional facts from all available resources, including the internet. Regarding their decision on CGT, advice and support from family members or friends was reported as less important, which may indicate that patients feel they decide—or are expected to decide—on a more rational and self-determined basis. Alternatively, the role of the physician may have been too dominant in the patient group studied, masking potential additional factors.

### Relevance and consciousness of decision-making for CGT

In contrast to the qualitative findings from Cook et al., many patients in our cohort saw a “decision to be made,” as the majority of patients responded that knowing the result will be important in their future life and planning [9]. Moreover, our patients seemed to decline CGT in order to avoid a “loss of autonomy” associated with the expectation that the CGT result could have an “unintended influence” on their future life and planning. Both concerns were associated with decision-making and were even more pronounced in patients with high baseline anxiety levels. In sharp contrast, Cook et al. reported that patients experienced the decision-making as “normative,” “automatic,” “not optional,” or “part of routine care,” possibly because these patients had to decide about CGT within a time frame of 24 h from diagnosis to intervention [9]. It is likely that these patients were already under the impression of the imminent primary treatment and had already switched to a more passive and submissive role defined by the upcoming surgery.

### Role of coping

While designing this trial, we assumed that coping strategies would have a major influence on the decision-making of our patients. Interestingly, for most coping strategies, we observed no substantial associations to interest in CGT. The exception was coping with “acceptance,” which was more frequently found in patients not interested in CGT, although it was not an independent predictor of the patients’ decisions. Coping with “acceptance” was negatively correlated with lower anxiety (*ρ* = –0.345) and depression levels (*ρ* = –0.290); it may facilitate decision-making by enabling better control of fearful expectations.

### Role of anxiety and depression in decision-making and decision regret

Schuermeyer and colleagues reported that up to 17% of patients regret their decision for CGT 3 months after testing [13]. Importantly, in this study, decision regret was not associated with a bad prognosis of CGT but was more frequently found in patients with higher anxiety or depression rates. A reason may be that some of these patients did not experience the expected feeling of reassurance after receiving a good prognosis or become “hopeful” after receiving a bad prognosis[9]. Our finding that anxiety is associated with the decision-making of our patients may correspond to the role of anxiety in decision regret: the more anxiety triggers interest in CGT, the more these patients may regret their decision in the future.

Our questions on the expected impact of CGT results on future social relations have not been addressed previously. Our results may indicate that patients opting for CGT hope to receive future support from friends and simultaneously fear social rejection by colleagues. More anxious patients, especially, seem to be more susceptible to these questions that may guide their decision about CGT.

### Strengths and limitations

In this prospective cross-sectional study, we investigated various environmental, psychological, and motivational factors that may contribute to decision-making about CGT in uveal melanoma patients. The prospective nature of this study, the high questionnaire return rate, the homogeneous treatment method, and our focus on individual patient concerns regarding CGT may contribute to the relevance of our data. A major limitation of this study is the single survey time point, which prevents us from drawing conclusions about the evolving psycho-social impact of the patient’s decision about CGT and their reflections on it.

### Conclusion

This study may contribute to our understanding of how patients manage their decision-making about CGT while being exposed to various preconditions, external influences, expectations, and concerns regarding their future. Although physicians’ advice and external information resources play a major role in patients’ decisions about CGT, concerns about the future and assumptions also influence their decision-making and should be considered during careful psycho-oncologic support.

Ongoing research on this issue should more profoundly involve patient-reported self-reflection on patients’ future concerns and furtherly investigate its potential interactions with informed consent regarding CGT.

## Data Availability

The datasets used and analyzed during the current study are available from the corresponding author on reasonable request.
